# Description of a new species of Ectinorus (E. spiculatus) (Siphonaptera, Rhopalopsyllidae) from Argentina and a review of the subgenus Ichyonus Smit, 1987

**DOI:** 10.3897/zookeys.124.1688

**Published:** 2011-08-18

**Authors:** Michael W. Hastriter, Richard D. Sage

**Affiliations:** 1 Monte L. Bean Life Science Museum, Brigham Young University, 290 MLBM, P.O. Box 20200, Provo, Utah 84602–20200, U.S.A.; 2 Museum of Vertebrate Zoology, University of California, Berkeley, California 94720, U.S.A.

**Keywords:** Argentina, Flea, *Ectinorus spiculatus*, *Ichyonus*

## Abstract

A new species, *Ectinorus spiculatus*, is described from *Phyllotis xanthopygus* (Waterhouse) and *Akodon iniscatus* Thomas from Neuquén Province, Argentina. Habitat characteristics are presented for the type locality. A change in status of four additional subspecies of the *Ectinorus* subgenus *Ichyonus* Smit is provided. *Ectinorus onychius onychius* Jordan and Rothschild, *Ectinorus onychius deplexus* Smit and *Ectinorus onychius angularis* Smit & Rosický were elevated to specific status. *Ectinorus (Ichyonus) onychius fueginus* was relegated as a junior synonym of the nominate species. *Phyllotis xanthopygus*, *Abrothrix olivaceus xanthorhinus*, *Loxodontomys micropus* Waterhouse, and *Euneomys chinchilloides* (Waterhouse) are new host records for *Ectinorus onychius*. A key to the three species of *Ichyonus* is included.

## Introduction

This study is a continuation of natural history studies of small mammals and their ectoparasites conducted in Neuquén and Río Negro Provinces, Argentina by the junior author (RDS). Among specimens collected during these studies, a new species of *Ectinorus* Jordan was discovered and is described herein. A synopsis of the genus *Ectinorus* is summarized in Hastriter and Sage (2009) and includes a comprehensive listing of known species and their distribution. Specimens of the subgenus *Ichyonus* Smit were also found in Neuquén Province which stimulated a further assessment of this enigmatic subgenus. Smit (1987) recognized four subspecies in the subgenus *Ichyonus* and his evaluation of these four taxa was based on relatively few specimens. The nominate subspecies comprised a good series of males and females, but only five specimens are representative of the other three subspecies. Of the latter three only males are known for two of them. With the availability of additional material, the status of taxa within this subgenus could be further evaluated. Systematic changes are addressed here. Including the new description that follows and a revision of available material representing *Ichyonus*, the genus is now comprised of 38 species [subgenera *Ectinorus* (34), *Ichyonus* (3) and *Panallius* Jordan (1)].

## Materials and Methods

Techniques for trapping small mammals and processing them for ectoparasites are outlined in detail in Hastriter and Sage (2009). Fleas were mounted on glass microscope slides in accordance with Hastriter and Whiting (2003). Dissections of male genitalia follow the procedures of Hastriter (2004). Images were prepared using an Olympus BX61 Compound Microscope, Olympus CC12 digital camera accompanied with an Olympus Microsuite™ B3SV program. Land marks used for flea measurements are described in Hastriter and Eckerlin (2003). Anatomical terms for flea anatomy are adapted from Rothschild and Traub (1971) and mammal nomenclature follows those of Wilson and Reeder (2005). Acronyms for repositories for “material examined” and type specimens follow:

**BMNH**	British Museum of Natural History, London

**BYU**	Monte L. Bean Life Science Museum, Brigham Young University, Provo, Utah

**CMNH**	Carnegie Museum of Natural History, Pittsburgh, Pennsylvania

**JCB**	Jean-Claude Beaucournu personal collection, Rennes, France

**MACN**	Museo de Ciencias Naturales “Bernardino Rivadavia” de la Ciudad de Buenos Aires, Republica Argentina

## Results

### Rhopalopsyllidae

**Key to the** Ectinorus **subgenus** Ichyonus **Smit**

**Table:** 

1	Males	2
–	Females (*Ectinorus deplexus* unknown)	4
2	Four stout setae on dorso-posterior margin of telomere and without subtending sinus; ventro-caudal hook on telomere extending upward less than ¼ the length of the telomere (Fig. 3). Upper portion of acetabulum at about mid-point of anterior margin of telomere (Fig. 3). Lobe on caudal margin of distal arm of ninth sternum lacking or only slightly indicated. Crochet hyaline and rounded at apex (Fig. 6); (see exception in diagnosis)	*Ectinorus onychius*
–	Seven to eleven stout setae on dorso-posterior margin of telomere; with distinct subtending sinus (Figs 1–2). Hook on telomere extending more than ¼ the length of the telomere. Upper margin of acetabulum either distinctly above or below middle of anterior margin of telomere. Lobe on caudal margin of distal arm well developed	3
3	Mesal surface of hind femur with row of 11–12 setae. Crochet expanded, rounded at apex with wide sclerotization along ventral margin; ventro-caudal margin with series of convoluted folds (fold similar but less apparent in Chilean specimens). Apex of ventro-caudal hook of telomere extending upwards more than half the length of telomere; upper margin of acetabulum far below middle of anterior margin of telomere (Fig. 1)	*Ectinorus angularis*
–	Mesal surface of hind femur with row of 17 setae. Crochet not expanded, but somewhat rectangular and truncate at apex; ventro-caudal angle with small lobe (Fig. 5). Apex of ventro-caudal hook of telomere not reaching half length of telomere; upper margin of acetabulum above middle of anterior margin of telomere (Fig.2)	*Ectinorus deplexus*
4	Anal stylet short, length twice width (2.1×) (Fig. 7)	*Ectinorus angularis*
–	Anal stylet longer, length greater than three times width (range: 3.2–3.6×, average: 3.5×) (Fig. 8)	*Ectinorus onychius*

#### Ectinorus (Ichyonus) angularis

Smit and Rosicky

http://species-id.net/wiki/Ectinorus_(Ichyonus)_angularis

Figs 1, 4, and 7

Ectinorus onychius angularis Smit and Rosicky 1972: 366; Smit 1987: 122; Beaucournu and Gallardo 1992: 100; Alarcón 2000: 13. stat. n.

##### Material Examined.

 Chile, [Magallanes Region]: Estancia Pudeto, W of Lago Sarmiento, [51°05'S, 73°00'W , ex *Akodon* sp.], 18 II 1969, M. Rozehnal (1 pair of paratypes) (BMNH).

##### Diagnosis.

**Figures 1–8.**
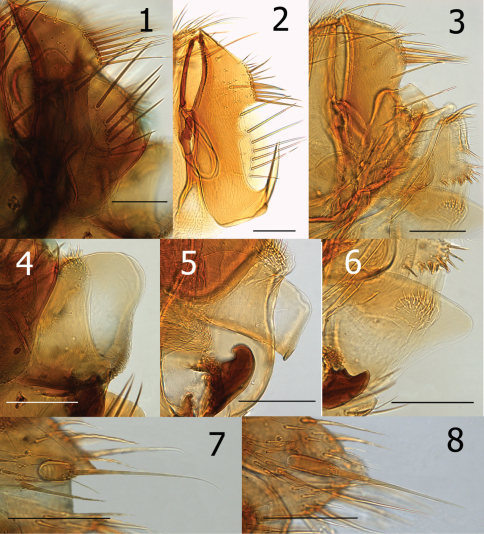
**1** *Ectinorus angularis*, telomere, paratype **2** *Ectnorus deplexus*, telomere, paratype **3** *Ectinorus onychius*, telomere, holotype **4** *Ectinorus angularis*, crochet, paratype **5** *Ectinorus deplexus*, crochet, holotype **6** *Ectinorus onychius*, crochet, holotype **7** *Ectinorus angularis*, anal stylet, paratype **8** *Ectinorus onychius*, anal stylet, paratype. Scale = 100µ

 Male distinguished from other species of *Ichyonus* by large lobular crochet that has a heavily sclerotized ventral margin and convoluted folds on the ventro-caudal margin (Fig. 4). The ventro-caudal hook of the telomere is also much longer and robust, its apex hooking upward well beyond middle of telomere (Fig. 1). This extends less than half the length of the telomere in other species. The upper portion of the acetabulum far below middle of anterior margin of telomere (Fig. 1). The single known female specimen may be distinguished from females of *Ectinorus onychius* by a much shorter anal stylet (*cf* Figs 7 and 8).

##### Remarks.

 Known only from a single collection in the extreme southern limits of Chile; little can be said of its host preferences. The type locality of *Ectinorus angularis* occurs at the lowest elevation of the three species of *Ichyonus* at ~40m. The female is indistinguishable from females of *Ectinorus onychius* with the exception of the much shorter anal stylet. The anal stylet on both sides of the single specimen examined appears the same. Two additional females (paratypes) reportedly exist in the Czechoslovak Academy of Sciences, Prague, but they could not be obtained for examination. Smit and Rosicky (1972) examined all three of the known females at the time of their description. Although they did not state that the anal stylet was similarly short in all three specimens, Smit (1987:124, fig. 263) subsequently illustrated the anal stylet of the allotype. Additional collections will refute or substantiate the value of this character.

#### Ectinorus (Ichyonus) deplexus

Smit

http://species-id.net/wiki/Ectinorus_(Ichyonus)_deplexus

Figs 1, 5 9

Ectinorus onychius onychius  Jameson and Fulk 1977:402 (mis-identification)
Ectinorus onychius deplexus Smit 1987: 120; Beaucournu and Gallardo 1992: 100; Alarcón 2000: 13. stat. n.

##### Material Examined.

 **Chile**, Santiago Province: La Parva, 3000m, ex *Eunomys noelli* (sic!) [*Euneomys noei*] = *Euneomys mordax* Thomas, Geo. Fulk (♂ holotype and ♂ paratype) (BMNH).

##### Diagnosis.

**Figure 9.**
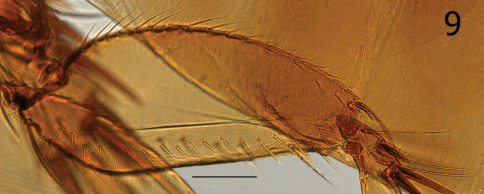
*Ectinorus deplexus*, paratype, hind femur. Scale = 100µ

 Female unknown. Setae on mesal surface of hind femur longer and more numerous (17) than other species (Fig. 9). Similar to *Ectinorus angularis* in the number of large setae on the caudal margin of the telomere, but distinguished by the much shorter length of the telomere from its apex to the upper margin of the acetabulum (Fig. 2).

##### Remarks.

 *Ectinorus deplexus* is known only from the type locality at an elevation that is more than three times that of the other two species of the subgenus *Ichyonus*. Elevational limitations may prove to be a factor in the distribution of other *Ichyonus* species. The two males are also notably larger (2.8mm) than males of *Ectinorus onychius* (2.1mm; n=11) and *Ectinorus angularis* (2.4mm; n=2).

#### Ectinorus (Ichyonus) onychius

(Jordan and Rothschild)

http://species-id.net/wiki/Ectinorus_(Ichyonus)_onychius

Figs 3, 6, and 8

Parapsyllus onychius Jordan and Rothschild 1923: 352; Dalla Torre 1924: 19; Del Ponte and Riesel 1939: 545.
Ectinorus onychius  (Jordan and Rothschild), Jordan 1942b: 11.
Ectinorus onychius onychius  (Jordan and Rothschild), Jordan 1942a: 434; Da Costa Lima and Hathaway 1946: 149; Smit 1955: 337 (♀ description); Johnson 1957: 142; Smit 1963: 427; Giménez, Ciccarelli and de la Barrera 1964: 129, 138, 139; Jameson and Fulk 1977: 402 (misdetermination, originally referred to *Ectinorus onychius deplexus*); Smit 1987: 116; Beaucournu and Gallardo 1988: 100; Beaucournu and Alcover 1989: 491; Beaucournu and Kelt 1990: 648, 665; Beaucournu and Gallardo 1992: 100; Alarcón 2000: 13; Beaucournu and del Carmen Castro 2003: 468. stat. n.
Ectinorus onychius fueginus Jordan 1942a: 434; *Ectinorus onychius fueginus* Jordan, Da Costa Lima and Hathaway 1946: 149; Johnson 1957: 142; Smit 1987: 121; Beaucournu and del Carmen Castro 2003 :468. syn. n.

##### Material Examined.

 **Argentina**, Chubut Province: Cholila, ex *Ctenomys haigi* Thomas, 23 I 1920, H.E. Box (♂ holotype); Cholila, ex *Akodon iniscatus* Thomas, 23 I 1920, H.E. Box (♂ paratype) (BMNH). Puerto Madryn, 6, 9 IV 1978, A. Kovacs (1♂, 1♀) (JCB). Mendoza Province: Puesto “Pugin", Algattolito (32°53'S, 67°18'W ), 620m, ex “rats", D.F. Giménez (♀) (BMNH). Neuquén Province: Laguna Blanca National Park, Locality 40, 1.97 km W, 3.84 km N Mellizo Sud, (39°2'27.5"S, 70°19'24.5"W ) inside clumps of “molle" (*Schinus polygama*) on sandy soil, 1290m, ex *Abrothrix olivaceus xanthorhinus*, 17 II 2006, R.D. Sage (♂, RDS-17963/F-276); Laguna Blanca National Park, Locality 74, 0.04 km E, 3.22 km S Cerro de la Laguna, (39°03'5.03"S, 70°22'30.42"W ), *Nassauvia* shrubland on dry slope above Laguna Blanca, south side of Península de la Laguna Blanca, 1285m, ex *Akodon iniscatus* (♂), 20 II 2007, R.D. Sage(♂, RDS-18370/F-282) (BYU); Laguna Blanca National Park, Locality 52, 0.27 km E, 3.93 km N Cerro de la Laguna, (38°59'9.84"S, 70°22'7.02"W ), grass scrubland (*Berberis* sp. and *Colletia* sp.) on east facing slope of rimrock, 1340m, ex *Euneomys chinchilloides* (Waterhouse) (♂), 27 VII 2007, R.D. Sage (2 ♀, RDS-18493) (MACN); Laguna Blanca National Park, Locality 76, 2.24 km W, 3.12 km S Cerro Mellizo Sud, (39°6'10.92"S, 70°19'33.42"W ), lava outcrops with *Colliguaja* sp., 1320m, ex *Phyllotis xanthopygus* (Waterhouse) (♂), 13 III 2007, R.D. Sage. (♀, RDS-18397) (BYU); Currhué Chico, ex *Akodon olivaceus* = *Abrothrix olivaceus* Waterhouse, 22 IV 1988, J.C. Beaucournu (♂); Lago Currhué, *Akodon longipilis* = *Abrothrix longipilis* Waterhouse, IV 1988, J.C. Beaucournu (♂); Lago Norquínco, ex *Abrothrix longipilis*, 1 V 1988, J.C. Beaucournu (2♀) (JCB). Río Negro Province: Trap line #6, Península Llao Llao, 0.4 km N Park Guard Station between Lagos Perito Moreno and Escondido, (41°02'54.7"S, 71°33'56.5"W ) in green bamboo/coíhue forest, 831m, ex *Loxodontomys micropus* Waterhouse (♀), 6 V 2005, R.D. Sage (♂, RDS-17339/F-278) (BYU); San Pedro, nr. Bariloche, ex “nest of *Rattus norvegicus*", 20 II 1954, J.M. de la Barrera (3♂, 1♀); San Pedro, nr. Bariloche, ex *Hesperomys* sp. [most likely a representative of either *Oryzomyz longicaudatus* or *Akodon longipilis*], 27 II 1954, J.M. de la Barrera (3♂, 2♀); San Pedro, nr. Bariloche, ex *Euneomys dabbenei* = *Ectinorus chinchilloides*, 8 VII 1953, J.M. de la Barrera (♀ paratype, “neallotype" on slide); Península of San Pedro, Bariloche, ex *Akodon varius neocenus* = *Akodon neocenus* Thomas [surely refers to *Akodon iniscatus*, since *Akodon neocenus* is only known to occur 150 km east of the Bariloche region, with *Akodon iniscatus* filling in the intervening area], 20 III 1960, J.M. de la Barrera (♀); Península of San Pedro, Bariloche, ex *Oryzomys longicaudatus philippii* = *Oligoryzomys longicaudatus* (Bennett), 20 III 1960, J.M. de la Barrera (♀); Bariloche, Nahual Huapi, ex *Rattus* sp., J.M. de la Barrera (1 male); El Bolsón, ex “nest of rodent", G. Topali & Don J. Szabo (♂); San Carlos de Bariloche, ex *Akodon olivaceus beatus* = *Abrothrix olivaceus*, 14 I 1965, J.M. de la Barrera (♀) (BMNH); San Pedro, nr Bariloche, ex *Oligoryzomys longicaudatus*, 26 II 1954, J.M. de la Barrera (♂); El Bolsón, 20 I 1961, Topal Gy. No. 58 (♀) (CMHN). Tierra del Fuego Province: Estancia Viamonte, ex *Belonopterus chilensis* = *Vanellus chilensis* Molina, 15 X 1931, P.W. Reynolds (*Ectinorus onychius fueginus* ♂ holotype, new synonymy herein) (BMNH). **Chile**, Aisén Region: Chico Aerodromo, ex *Akodon xanthorhinus* = *Abrothrix olivaceus xanthorhinus*, III 1987, J.C. Beaucournu (Kelt-3976, 3♂, 1♀); Puerto Ibanéz, El Salto, ex *Akodon longipilis* = *Abrothrix longipilis*, III 1987, J.C. Beaucournu (Kelt-3563, ♂, ♀) (BYU).

##### Diagnosis.

 *Ectinorus onychius* males differ from either *Ectinorus angularis* and *Ectinorus deplexus* by the lack of a strong lobe on the ventro-caudal margin of the distal arm of the ninth sternum, by fewer strong setae on the caudal margin of the telomere with only 4 (sometimes 5) (Fig. 3), and the hyaline crochet is longer than wide and round on the apex (without sclerotized margins or special feature at ventro-caudal margin) (Fig. 6). Note: the crochet of *Ectinorus onychius* specimens from Aisén Region, Chile is more similar to the single exemplar of *Ectinorus angularis* in Magallanes Region, Chile than the more northern populations of *Ectinorus onychius* in Chubut, Neuquén, and Rio Negro Provinces, Argentina. Never-the-less, populations further north in Argentina and those in Aisén Province, Chile are clearly distinct from *Ectinorus angularis* by the greater superior position of the acetabulum on the telomere. Female distinguished from *Ectinorus angularis* by the much longer anal stylet (*cf*. Figs 7 and 8)

##### Remarks.

 There is insufficient morphological evidence to support the erection of *Ectinorus onychius fueginus* to full specific status, nor to recognize this single specimen as a subspecies. It is considered a junior synonym of *Ectinorus onychius* from which it is indistinguishable. Our taxonomic re-interpretation extends the geographic range of *Ectinorus onychius* from northeastern Mendoza Province to the extreme southern province of Tierra del Fuego, and from the Atlantic coast (Puerto Madryn) to steppe habitat at the Argentina/Chilean border. Additional collections in the southern parts of the Monte phytogeographic biomes of Mendoza/La Pampa/Río Negro Provinces and in the south-central parts of the Patagonian region are needed to establish the distribution and true range of this species. The finding of the record on a plover, [*Belonopterus chilensis* = *Vanellus chilensis*] (Tierra del Fuego Province) is certainly accidental and is the only known record of the subgenus found on an avian host. Representatives of *Ichyonus* appear on many small sigmodontid rodents and without specificity. There is only one report of this species on a non-sigmodontid rodent, i.e., on the histricomorphid genus *Ctenomys*. The nearly total absence of this flea in our extensive study of ectoparasites of species of *Ctenomys* would suggest this is an accidental association. In addition to hosts previously reported on *Ectinorus onychius* (in lit.), our findings on *Phyllotis xanthopygus*, *Abrothrix olivaceus xanthorhinus*, *Loxodontomys micropus*, and *Ectinorus chinchilloides* are new host records. The reference to *Abrothrix xanthorhinus* as a subspecies of *Abrothrix olivaceus* is our attempt to identify the distinct race of this enigmatic sigmodontid complex. This flea is clearly more dependent on terraine, habitat, elevation, and microclimatic conditions than on host specificity.

A single specimen (RDS-17339) was collected from *Loxodontomys micropus* in the region of the Valdivian, evergreen rainforest. The specific site is in a mature forest of *Nothofagus dombeyi* (Mirb.) (“coihue”) with a dense understory of the bamboo *Chusquea culeou* Desvaux.(“caña coihue”) on a south-facing hillside (Fig. 24). There were many fallen and rotting trunks of the “coihue” on the ground, and a thick leaf litter comprised mostly of the bamboo leaves. The soil is dark in color and rich in humus. The environment is cool and moist. *Loxodontomys micropus* was the third-most abundant of the six rodent species in this habitat, with *Abrothrix longipilis* being the most common species (40 percent of the total collected at this site) from which *Ectinorus onychius* was not collected. The second specimen (RDS-18370) was collected from *Akodon iniscatus* on the large peninsula that juts into Laguna Blanca in LBNP. The peninsula has been protected from livestock grazing for 15 years and has a comparatively very dense development of the Patagonian steppe vegetation. In particular the eastern (leeward) side of the peninsula is densely covered with spiny shrub *Nassauvia glomerulosa* D. Don (“uña de gato”) and bunch grasses. The soil consists of fine, windblown sand, with little organic matter. The Laguna Blanca area is cool, dry, and strong winds are frequent. On this protected peninsula, *Akodon iniscatus* is the most abundant species, followed by *Eligmodontia morgani* J.A. Allen. Two additional specimens (RDS-18493) were collected from, *Euneomys chinchilloides* along the edge of a black basalt rimrock with a dense growth of the Patagonian steppe shrub *Colletia hystrix* (Clos.) (“espino negro”) and large bunch grasses (Fig. 25). The ground consisted of blocks of the broken basaltic rock and wind-blown sand. *Phyllotis xanthopygus* was the more common of the five species of rodents trapped in this habitat.

Measurements of the anal stylet of eight specimens were conducted. The range of their length was 58–71µ (average: 63µ) and width was 16–20 µ (average: 18µ) with an average ratio of 3.5× (length:width). This ratio is substantially greater than that of the single female of *Ectinorus angularis* (2.1×). It is doubtful that the shorter anal stylet is an anomalous condition, since the stylet on both sides are similarly short.

#### Ectinorus spiculatus

Hastriter and Sage sp. n.

urn:lsid:zoobank.org:act:C739CAB5-FAA9-4D33-8266-D7C5827DCA9F

http://species-id.net/wiki/Ectinorus_spiculatus

Figs 10 [Fig F4] 23 

##### Type Material.

 **Argentina**, Neuquén Province:, 1 km SSW from Route 40 on dirt road to Estancia Llamuco (38°44'1.2"S, 70°17'55.26"W ), vegetation on sandy soil with basaltic rimrock, 1074m, ex *Phyllotis xanthopygus* (♀), 14 IV 2008, R.D. Sage, Holotype ♂ (RDS-18861); Laguna Blanca National Park, Locality 76, 2.24 km W, 3.12 km S Cerro Mellizo Sud, (39°6' 10.92"S, 70°19'33.42"W ), lava outcrops with *Colliguaja* sp., 1320m, ex *Phyllotis xanthopygus* (♂), 14 III 2007, R.D. Sage, allotype ♀ (RDS-18407); same data as allotype except ex *Akodon iniscatus* (♂), 13 III 2007, paratype ♀ (RDS-18403). Holotype and allotype are deposited in the Museo de Ciencias Naturales “Bernardino Rivadavia" de la Ciudad de Buenos Aires, Republica Argentina; paratype ♀ deposited in the Monte L. Bean Life Science Museum, Brigham Young University, Provo, Utah, U.S.A.

##### Diagnosis.

 Males key to *Ectinorus hertigi* (Johnson) in Smit’s (1987:78) key, while females key to *Ectinorus barrerai* Jordan. Morphologically the male is closely allied with *Ectinorus hertigi* but may be distinguished from it and all other species of the subgenus *Ectinorus* by the bilobed apex of the basimere and details of the aedeagus (Figs 13, 16). The presence of seven segments in the labial palpus of females (male with five) is the basis for its similarity with *Ectinorus barrerai*; however, their similarity is limited. If one continues onward in the key using five segments in the labial palpus (versus 6 to 8), females key out to *Ectinorus hertigi* also. Females share many similarities with *Ectinorus hertigi* (few with *Ectinorus barrerai*) for which they may be separated by an oblique flattened region of the spermatheca at the cribriform area and a very long bursa copulatrix that is reflected postad in a semi-circular arc (Fig. 23).

##### Description.

**Figures 10–13.**
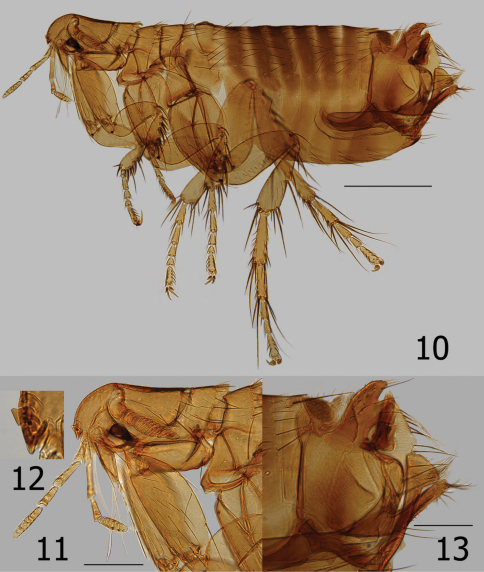
*Ectinorus spiculatus* sp. n., male, holotype **10** Overview **11** Head, pronotum and forecoxa **12** Enlargement of frontal tubercle (insert) **13** Terminal segments. Scale Fig. 10 = 500µ; Figs 11 and 13 = 200µ

**Figures 14–16. F4:**
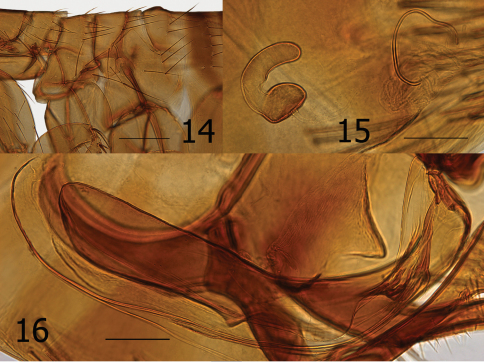
*Ectinorus spiculatu*s sp. n. **14** Male, holotype, thorax and metepimeron **15** Female, allotype, spermatheca and bursa copulatrix **16** Male, holotype, aedeagus. Scale Fig. 14 = 200µ; Figs 15–16 = 100µ

 Chaetotaxy and structural references include only one side of specimen. Head (Figs 10–12). Frons evenly rounded; thickened throughout. Frontal tubercle quadrate; capsule heavily sclerotized but thin caudad. Two placoids between frontal tubercle and sclerotized antennal suture. Eye large, darkly pigmented, sinuate. Ocular setae four; laterals large, middle two much smaller. Tentorium clearly visible anterior to eye. Preantennal setae; one near oral angle, two (large and small) anterior to eye. Third segment of maxillary palpus shorter than others; maxilla acutely sharp. Five segmented labial palpus extending to apex of coxa, apical two segments twice length of either second or third segments; apex blunt with array of fine setae. Antennal scape with apical row of six fine setae; pedicel with three minute dorsal setae; clavus extending onto prosternasome. Post-antennal area with four rows of setae (1, 1, 1, 6 plus intercalaries; female with only two minute setae anterior to main row). Two placoids; occipital groove moderately deep. Row of 18 setules along dorsal margin of antennal groove. Genal lobe bluntly rounded with three small apical setae; five larger marginal setae below eye. Thorax (Figs 10, 11, 14). Pro-, meso-, and metanota each with two rows setae. Eleven to 12 pseudosetae under mesonotal colar. Dorsal apex of metanotum curled downward. Cervical link-plate truncate at apex. Prosternasome grooved for retention of antennal apex; without setae. Mesepimeron with four setae and mesepisternum with two; mesosternum heavily sclerotized along ventral margin with incomplete suture between mesepisternum. Pleural rod bifurcate dorsally. Lateral metanotal area with two large, two small setae. Pleural arch and ridge well developed. Metepisternum and metasternum, fused into one; one large seta. Furca long and delicate. Metepimeron with two vertical rows of setae; anterior with two (dorsal minute), posterior of three (same arrangement in female). Legs (Figs 10, 19). Fore coxa with 28 lateral setae; one long seta at posterior margin. Oblique break mid coxa indicated only at ventro-caudal margin. Two guard setae at femoral-tibial joints; lateral of two long equal on fore femur; shorter on mid and hind femora. Fore and mid femora with two lateral rows of setae; hind femur with single lateral row of 12 setae. Lateral sculpturing of hind femur very fine. Margin of fore, mid, and hind tibiae with 5, 6, and 6 dorsal notches, respectively. Number of setae in respective dorsal notches: fore tibia (beginning with proximal notch) (2, 2, 3, 2, 3), mid tibia (2, 2, 2, 3, 2, 3), hind tibia (2, 2, 2, 2, 2, 3). Lateral setae of each tibia, respectively (6, 6, 8). Inner (mesal) surface of hind tibia adorned with spicules. First hind tarsus with three long setae; two extending to and one extending beyond segment three. Second hind tarsus with two setae extending beyond distotarsomere. Distotarsomeres with four pair lateral plantar bristles; apical pair smallest. Pre-apical plantar bristles two; one small, one larger. Ungue symmetrical. Unmodified Abdominal Segments (Fig. 10). Dorsum of tergum I heavily sclerotized with distinct hump (absent in female); anterior lateral margin thick and sclerotized. Two rows setae. Terga II–III with two rows setae; terga IV–VII with single row. Ventral most setae of terga II–VII not extending below level of round spiracles. Single antesensilial bristles extending from pedestal beneath apical flange of tergum VII. Sternum II with lateral patch of 7–8 small setae. Sterna II-VII with single rows of setae (1, 2, 3, 3, 3, 3). Modified Abdominal Segments (Male) (Figs 10, 13). Sensilium with 17 sensilial pits; surrounded by wide sclerotized area bearing single seta on caudal margin. Spiracle VIII vermiform, curved upward with three small setae dorsad. Tergum VIII large and highly specialized; lateral and apical surfaces with coarsely reticulated pattern. Tergum VIII envelops basal portion of basimere while curling under and behind apical portion of basimere and telomere to form an unusual conical sharp lobe. Caudal margin is adorned with eleven long setae; ventro-caudal margin with two long setae and smaller marginal setae cephalad. Sternum VIII with lateral row of eight long setae; ventral apex with thick incrassation. Dorsad to incrassation extends a moderately sharp projection. Apex of basimere (tergum IX) with two asymmetrical lobes divided by a deep sinus. Dorsal lobe of basimere with numerous setae; ventral lobe with two stout setae. Robust *processus basimeris ventralis* present; group of stout setae at apex. Length of telomere more than five times width; bluntly rounded at apex, sides parallel. Numerous small setae line margin. Manubrium tapered, curving upward to acute point. Lateral portion of basimere with triangular, darkly sclerotized, caudally directed structure (Fig. 16). (A patch of fine setae are present on each side and appear to be present on a lobe ventrally located on the ventral margin of tergum VIII and may be associated with triangular sclerotization above. (Without dissection of genitalia, this anatomy could not be deciphered for certain). Distal arm of sternum IX long with parallel sides, expanding at tip; lateral setae present on upper third. Notable group of 9–10 long setae on caudally expanded lobe. Vestigial tendon of sternum IX affixed to apical sclerotization of sternum VIII. Aedeagus (Figs 10, 13, 16). Similar to that of *Ectinorus hertigi*, but median dorsal lobe greatly reduced and lateral lobes expanded. Dorsal armature immense (seen behind basal portion of telomere sandwiched between conical lobe of tergum VIII, Fig. 13), ventral armature reduced. Sclerotized inner tube long, slightly curved ventrad; with annular ring at midpoint. Aedeagal apodeme bluntly rounded at apex; penis rods barely extend beyond apex of apodeme. Modified Abdominal Segments (Female) (Figs 15, 17, 18, 23). Seventh sternum with lateral row of five setae; caudal margin entire with ventral margin incised, creating an indistinct rounded ventral lobe. Single antesensilial bristle arising from strong pedicel. Tergum VIII with group of eight setae above spiracle VIII. Spiracle VIII vermiform, slightly ballooned at base. Lateral row of six long setae on tergum VIII; marginal group of 20 plus setae at apical margin. Sternum VIII with apical rounded lobe, without setae. Sensilium with broad sclerotized ring; 16 sensilial pits. Anal stylet with apical long seta plus one seta longer than anal stylet. Length of anal stylet twice width. Hilla twice length of bulga; hilla approximate width of bulga. Bulga flattened on cribriform region; cribriform area not protruding into bulga. Bursa copulatrix extremely long; curved caudally in circular arc.

Length: Male holotype: 2185µ; female allotype: 2533µ; and female paratype: 2175µ.

**Figurs 17–23.**
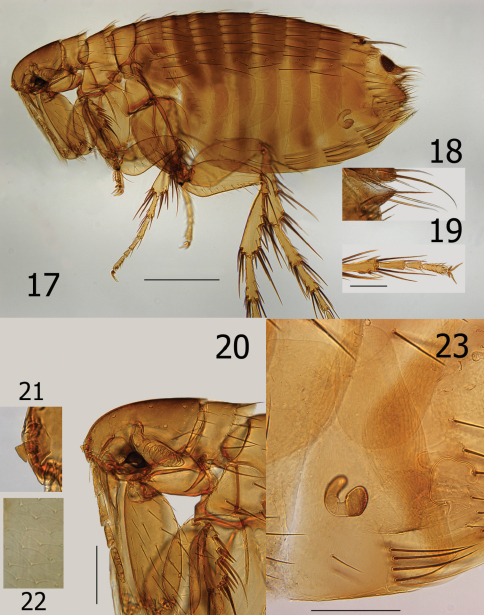
*Ectinorus spiculatus* sp. n., female, allotype **17** Overview **18** Dorsal and ventral anal segments **19** Hind tarsal segments **20** Head, pronotum, and forecoxa **21** Enlargement of frontal tubercle (insert) **22** Spiculated medial surface of hind tibia (insert) **23** *Ectinorus spiculatus* sp. n., female, paratype, seventh sternum, spermatheca, and bursa copulatrix. Scale Fig. 17 = 500µ; Figs 18–20 and 23 = 200µ

##### Etymology.

 The specific epithet *spiculatus* is derived from the characteristic presence of spicules on the mesal surface of the hind tibia.

##### Remarks.

 The single male and two females were all collected from different host specimens. The authors feel confident that both sexes belong to the same taxon for the following reasons: 1) Both male and female have spicules on the mesal surface of the hind tibiae, 2) both sexes have very similar head chaetotaxy and shape of the genal lobe, 3) the second tarsal segment possesses three long setae, two of which extend beyond segment four, 4) a pair was collected at the same locality (Laguna Blanca National Park) and the other female within close proximity, within 35 km, 5) the male at one locality and female from the other were from the same host species (*Phyllotis xanthopygus*), and 6) terraine, habitat, and elevations for both localities were nearly the same.

The holotype (RDS-18861) was collected from *Phyllotis xanthopygus* along the edge of a rimrock of a dark-red basaltic flow from a nearby, unnamed cinder cone (Fig. 26). Deep drifts of unconsolidated, wind-blown sand, filled the fissures in this broken-rock habitat. A dense growth of *Colliguaja integerrima* Gillies & Hook (“coliguay”) and bunch grasses were the dominant plants. The area was cold, dry, and at times very windy. Only *Phyllotis xanthopygus* and an undescribed species of *Ctenomys* (“tuco-tuco”) were trapped at the type locality. It should be noted that *Ectinorus lareschaei* Hastriter and Sage, 2009 was also collected from the same host specimen as this holotype. Paratypes RDS-18403 and RDS-18407, were collected from *Akodon iniscatus* and *Phyllotis xanthopygus*, respectively, in Laguna Blanca National Park at the southern edge of the lava flow forming the cinder cone volcano, Cerro Mellizo Sud. Deep sandy soil fills in the small fissures in the lava flow and there is a sparse growth of the Patagonian steppe vegetation, mostly bunch grasses and smaller shrubs such as *Ctenomys integerrima* and *Nassauvia glomerulosa*. *Phyllotis xanthopygus* was the more common of the five small mammals trapped here, including the mouse opossum *Thylamys pallidior* (Thomas).

**Figure 24.**
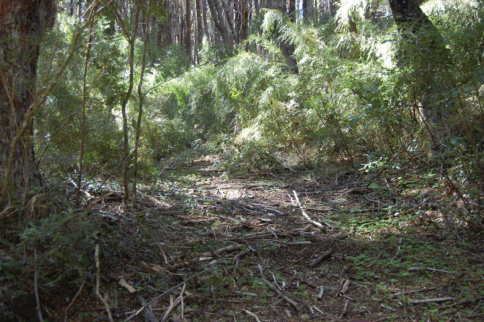
Habitat from which *Ectinorus onychius* was collected from *Loxodontomys micropus*.

**Figure 25.**
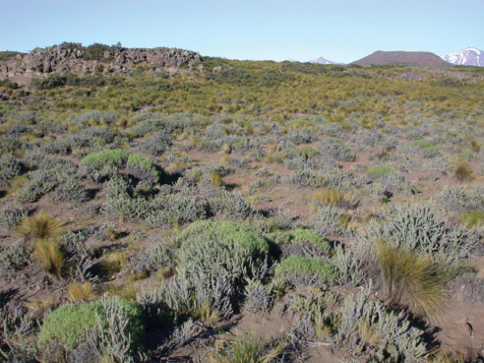
Habitat from which *Ectinorus onychius* was collected from *Euneomys chinchilloides*.

**Figure 26.**
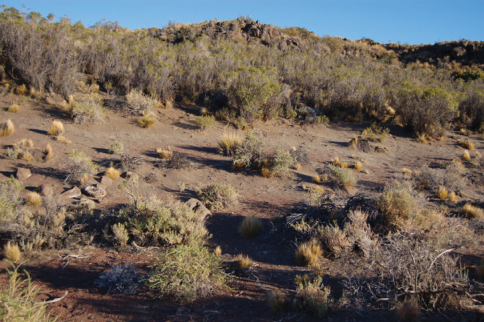
Habitat from which *Ectinorus spiculatus* holotype was collected from *Phyllotis xanthopygus*.

## Supplementary Material

XML Treatment for Ectinorus (Ichyonus) angularis

XML Treatment for Ectinorus (Ichyonus) deplexus

XML Treatment for Ectinorus (Ichyonus) onychius

XML Treatment for Ectinorus spiculatus

## References

[B1] Alarcón ME (2000) Estado actual del conocimiento de los siphonapteros presents en Chile (Insecta: Siphonaptera). Gayana 64(1): 1–17

[B2] Beaucournu JC Alcover JA (1989) Puces récoltées dans la province de Neuquén (Argentina); description de 4 nouveaux taxa (Insecta, Siphonaptera). Anales de Parasitologie Humaine et Comparee. 64 (6): 489-505 10.1051/parasite/1989646489 2624378

[B3] Beaucournu JC del Carmen-Castro D (2003) Contribution à un inventaire des puces d’Argentine (Insecta – Siphonaptera). Beitrage zur Entomologie 53 (2): 449-479

[B4] Beaucournu JC Gallardo MN (1988) Puces nouvelles d’Argentine (Insecta, Siphonaptera). Revue Suisse de Zoologie 95 (1): 99-112

[B5] Beaucournu JC Gallardo MH (1992) Catalogue provisoire des puces du Chile (Insecta; Siphonaptera). Bulletin de la Société Française de Parasitologie 10 (1): 93-129

[B6] Beaucournu JC Kelt DA (1990) Contribution a la faune du Chile: puces nouvelles ou peu connues de la partie sud (Insecta, Siphonaptera). Revue Suisse de Zoologie 97 (3): 647-668

[B7] Costa Lima A da Hathaway CR (1946) Pulgas: Bibliografia, catálogo e hospedadores. Monografias do Instituto Oswaldo Cruz, No. 4: 1-522 pp.

[B8] Dalla Torre CG (1924) Aphaniptera. Sonderabdruck aus den Berichten des naturwissenschafilich-medizinischen Vereines in Innsbruck 39: 1-29

[B9] Del Ponte E Reisel MA (1939) Notas sobre “Siphonaptera” argentines. II. Primera lista de species. Physis, Revista de la Sociedad Argentina de Ciencias Naturales, Buenos Aires 17: 543-551

[B10] Hastriter MW Eckerlin RP (2003) *Jellisonia painteri* (Siphonaptera: Ceratophyllidae), a new species of flea from Guatemala. Annals the of Carnegie Museum 72: 215-224

[B11] Hastriter MW Whiting MF (2003) Siphonaptera (Fleas). In: Resh VH Carde R (Eds) Encyclopedia of Insects, Academic Press, 1040–1044

[B12] Hastriter MW (2004) Revision of the flea genus *Jellisonia* Traub, 1944 (Siphonaptera: Ceratophyllidae). Annals of the Carnegie Museum 73 (4): 233-257

[B13] Hastriter MW Sage RD (2009) A description of two new species of *Ectinorus* (Siphonaptera: Rhopalopsyllidae) from Laguna Blanca National Park, Neuquén Province, Argentina. Proceedings of the Entomological Society of Washington 11 (3): 581-597 doi: 10.4289/0013-8797-111.3.581

[B14] Jordan K (1942a) On Siphonaptera collected by Dr. J.M. de la Barrera in the province of Mendoza during 1939. Revista del Instituto Bacteriologico “Dr. Carlos G. Malbran” 10 (4): 401-460

[B15] Jordan K (1942b) On *Parapsyllus* and some closely related genera of Siphonaptera. EOS, Revista Española de Entomologia 18: 7-29

[B16] Jordan K Rothschild NC (1923) On the genera *Rhopalopsyllus* and *Parapsyllus*. Ectoparasites 1: 320-370

[B17] Rothschild M Traub R (1971) A revised glossary of terms used in the taxonomy and morphology of fleas (reprinted from G.H.E. Hopkins and M. Rothschild, An Illustrated Catalogue of the Rothschild Collection of Fleas (Siphonaptera) in the British Museum (Natural History). Vol. 5, 8–85

[B18] Smit FGAM (1987) An illustrated catalogue of the Rothschild collection of fleas (Siphonaptera in the British Museum (Natural History), Vol. VII, Malacopsylloidea (Malacopsyllidae and Rhopalopsyllidae), Oxford University Press, British Museum (Natural History), Oxford and London, 5 plates, 380 pp.

[B19] Smit FGAM Rosicky B (1972) Some Siphonaptera from Chile. Folia Parasitologica (Praha) 19: 365-368 4670844

[B20] Wilson DE Reeder DM (2005) Mammal species of the world. A taxonomic and geographic reference. 3rd Ed., The Johns Hopkins University Press, Baltimore

